# Experiment design driven FAIRification of omics data matrices, an exemplar

**DOI:** 10.1038/s41597-019-0286-0

**Published:** 2019-12-12

**Authors:** Philippe Rocca-Serra, Susanna-Assunta Sansone

**Affiliations:** Oxford e-Research Centre, Department of Engineering Science, University of Oxford, 7 Keble Road, Oxford, OX1 3QG United Kingdom

**Keywords:** Communication and replication, Data publication and archiving

## Abstract

We outline a principled approach to data FAIRification rooted in the notions of experimental design, and whose main intent is to clarify the semantics of data matrices. Using two related metabolomics datasets associated to journal articles, we perform retrospective data and metadata curation and re-annotation, using community, open, interoperability standards. The results are semantically-anchored data matrices, deposited in public archives, which are readable by software agents for data-level queries, and which can support the reproducibility and reuse of the data underpinning the publications.

The scientific, economic and societal impact of research depends upon access to and reuse of the methods and data generated in every publication, and the utility of genomic research depends increasingly upon access to appropriately curated phenotypic data^1^. For scalable, effective and trustworthy data-driven science, we need to ensure that data are Findable, Accessible, Interoperable and Reusable (FAIR) by humans as well as by machines. Since their publication in 2016^2^, the FAIR Principles of data management and stewardship have become pervasive in discussions, policies and implementations in and around technological and social infrastructure for research data^3,4^.

The principles put specific emphasis on enhancing the ability of machines to find, access and process data, in addition to supporting their interoperability and reuse by individuals.

Responding to the invitation to make a rose dataset FAIRer^5^, we present a principled approach to data FAIRification, which focuses on the clarity of the statistical results. We showcase its application using the work by Raymond *et al*.^6^ on a targeted metabolite profiling study of strain-related chemical signatures of the rose fragrance. Our starting point was the human-understandable data provided by the authors as a supplementary table. Using community, open, interoperability standards, available from FAIRsharing^7^ (https://fairsharing.org), we performed the retrospective curation and re-annotation of the data matrices, disambiguating them using the experimental design information. To assess inter-experiments agreement, we applied the same procedure to a second data source^8^, which is an early work of the same group on the same varieties of rose and plant parts. The results are served in an open syntax and fully documented, as well as executable data science project, with jupyter notebooks. In the following sections, we detail the process needed to transform typical supplementary tables into machine readable information to enable data-level queries, and support the reproducibility and reuse of the data.

## The FAIRification Process

**Fig. 1:**
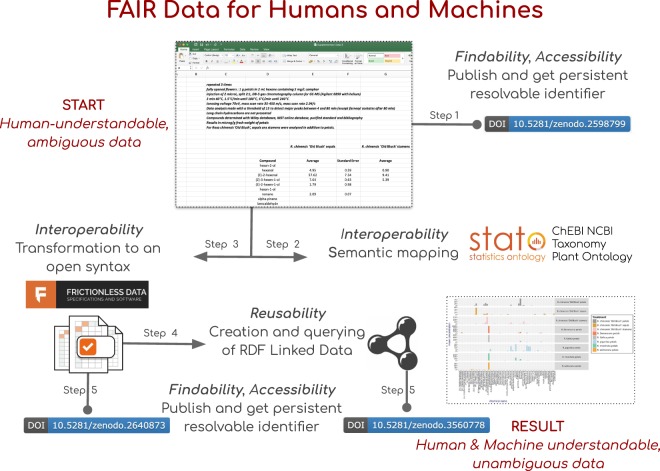
Summary and overview of the steps undertaken to make the targeted metabolite profiles of the Rose scent datasets FAIRer, and how each one address one of more of the FAIR elements. Step 1: made the initial spreadsheet table discoverable and citable, assigning an open license (CC-BY 4.0). Step 2: regularized the three dimensions of the matrix (which represent the metabolites, the treatments, and the quantitation type), by unpacking the information held in the column header, replacing free text with ontology terms, disambiguating the experimental design and clarifying the measurement performed. Step 3: used the Frictionless Tabular Data Package to describe the table headers in JSON format, documenting the transformation in a jupyter notebook. Step 4: performed a conversion to Linked Data, plotting the metabolite measurements using visualization libraries. Step 5: made the FAIRified outputs discoverable and citable, assigning an open license (CC-BY 4.0).

In the first article^6^, the “Supplementary Data Table 3” is a spreadsheet that collates the mean concentrations of 61 molecular compounds measured in different parts of the rose flower, in six distinct genotypes. Whilst this table is understandable to a human audience, it is not particularly suited to consumption by software agents, falling short on several of the FAIR Principles. In Fig. 1, we summarize the steps undertaken to make this dataset FAIRer. To address findability and accessibility of our work, we uploaded all relevant files to the Zenodo repository and assigned these artefacts an open license (CC-BY 4.0). Using the Digital Object Identifier (DOI) minted by Zenodo, the initial spreadsheet table^9^ and the associated FAIRified outputs^10,11^ are more discoverable and also formally citable. To address the interoperability and reusability of the data, we applied several steps. We exposed the semantics hidden behind the column headings in the spreadsheet by identifying the main types of tabulated entities and their relationships; we marked-up all entities with persistent resolvable identifiers to enhance dataset connectivity. We then regularized the matrix using a well-established syntax; and lastly, we performed a conversion to Linked Data.

### Semantic mapping

Conceptually, matrices of results are data cubes, or hypercubes, where the information is organized according to three (or more) dimensions, respectively. To be FAIR, the entities these dimensions hold must be unambiguously reported. In our case, the matrix defines three dimensions representing: i) the metabolites (or, more generally, molecular entities), ii) the ‘treatments (or experimental conditions and corresponding biomaterials and bioassays), and iii) the quantitation type (or generally, measurements). In the original spreadsheet table, the metabolites were reported using free text, which, in most instances, matched their common chemical name. Such practice is known to cause issues due to its imprecision. To address this point, we accessed CHEBI^12^ programmatically through its LibChebi library^13^ (https://github.com/libChEBI/libChEBIpy) and retrieved relevant CHEBI identifiers and InChI strings^14^, which are the community-developed, non-proprietary textual identifiers for chemical entities. Metabolites names were thus augmented with these unambiguous codes.

As a next step, we ‘unpacked’ the information held in the column header and semantically anchored it. Starting with the biological materials, originally denoted with composed terms such as “*R*. *chinensis ‘Old Blush’ sepals”*, we disambiguated the taxonomic name of the cultivar from the anatomical part using terms and identifiers from the NCBI Taxonomy^15^ and Plant Ontology^16^ respectively. The use of established and community-driven terminologies, which are used by a number of public resources, offer higher potential for data discovery and interoperability.

This initial semantic mapping, however, is just the first step towards FAIRer data. The values reported in these columns headers are in fact the *factor levels* of 2 *independent variables*, which need to be properly marked up and made explicit. To better explain this point, we ask the reader to consider the overall design of this experiment and the hypothesis being tested. These biological materials have been selected on purpose, “by design”, to allow a comparison between parts of the plant, and across cultivars in the same tissue type (sepals in this instance). Therefore, basing data reporting on study design concepts provides a *principled way* for organizing the description of such data matrices. Emerging terminologies, such as the STATistics Ontology (STATO; 10.25504/FAIRsharing.na5xp), are essential to unambiguously express and semantically type these notions. In our case, a *factorial design* was recognized, with two *independent variables* identified, namely the rose variety and the organism part, which are both categorical variables with six and three discrete *factor levels*, respectively. In such a context, 18 theoretical *factor combinations* are possible, as determined by the result of the cross product (cartesian product) for the two sets of variables levels, with each combination identifying a possible *statistical treatment*. Since only eight out of eighteen are reported, we conclude this is a *fractional factorial design*.

Making such notions explicit clarifies and disambiguates the intent of the experimentalists and delivers clearer and more reusable datasets. However, not all metadata models are capable of representing such information with sufficient granularity. For example, a number of models implemented by major public genomics databases have no dedicated objects to represent *study factors* and their values; variables are implicitly declared as sample attributes, and therefore the database cannot explicitly enable queries on treatment groups and their sizes.

The last dimension of the data cube corresponds to the *quantitation types*: two measurements were identified for each of the experimental conditions: average and standard error. To anchor those in a semantic framework, we have also used STATO to replace the string *average* with the class *sample mean*, which corresponds more specifically to the notion of *arithmetic mean*, and the field header s*tandard error* with the class *standard error of the mean*. The *sample mean* measurement, as formally defined by the STATO class, should be reported with the size of the sample over which the calculation is performed. Although incomplete, some of this information was provided by the authors in the “Reporting Summary” file, part of the Supplementary Information available from Raymond *et al*.^6^. It seems that for each treatment, a single biological material was prepared and assayed three times on the same analytical platform. Therefore, the computed sample mean can only be used to estimate the variability of the measurement technique, not the biological variability. Once again, reporting such critical information accurately is essential to data analysts and statisticians for them to confidently apply methods and software agents to process the data, for instance, to run a workflow with parameters set for specific classic univariate analysis, such as *2-way ANOVA*.

### Open syntax

Having clarified the semantics, the next step was aimed at ensuring the long term preservation of data matrices. We used the Frictionless Tabular Data Package, a simple container format used to describe and package a collection of data (https://frictionlessdata.io/data-packages). The package provides a description of table headers using a JavaScript Object Notation (JSON) format, a popular open-standard representation, used to validate the tabular data themselves, provided alongside, as comma or tab separated values. The transformation is fully documented in our jupyter notebook (https://github.com/proccaserra/rose2018ng-notebook/blob/master/notebooks/0-rose-metabolites-Python-data-handling.ipynb).

### Linked data

The last step of our FAIRification process is the creation of Linked Data, a method of publishing structured data so that it can be interlinked with other resources. The Resource Description Framework (RDF) is one of the key ingredients of Linked Data: it provides a generic graph-based data model for describing data that can be queried using the SPARQL language. The RDF representation, which relies on terms from OBO Foundry ontologies^17^, enables queries such as “Retrieve study predictor variables and their levels” and “What is the sample size used to compute the mean?”, therefore supporting study results review and assessment. As shown in the jupyter notebooks available as part of the code released for this work, the metabolite measurements themselves can be plotted using popular visualization libraries (Python plotnine or R ggplot2) from either a SPARQL query over the RDF representation or from the data package directly.

### Integration and preservation

To further demonstrate the value of such study design driven data representation, we applied a similar FAIRification process to the supplementary material from an earlier work by the same group^8^. This also helped to assess inter-experiment agreement, as both studies used the same varieties of rose and plant parts. However, we had to modify our annotation pipeline to extract the metabolite profiles, not just from spreadsheets but also from PDF tables, adding an extra step to our process. Such additional work is quite common when FAIRifying data retrospectively. The results of this comparison, also released via Zenodo^18^, can be visually explored using the aforementioned graphic grammar compatible libraries. A Venn diagram and an Upset plot^19^ provide a visual overview of the metabolites shared between the two studies, and are available along with the executable code used to generate them. Lastly, we produced a study description file, in ISA-Tab format^20^, which references the Tabular Data Packages representing the results held in data matrices. The ISA file is suitable for deposition to MetaboLights^21^, a public repository for metabolomics data recommended by several journals (10.25504/FAIRsharing.kkdpxe).

The code and data associated with this project are archived in Zenodo, as detailed below:

- Rose scent FAIRification project code release^22^.
- Associated Material to “The Rosa genome provides new insights into the domestication of modern roses” publication^9^.
- GC-MS data from the ‘Rose Genome’ available as Frictionless Tabular Data Package^10^.
- RDF Linked Data representation of GC-MS data from the ‘Rose Genome’ article^11^.
- Comparison of GC-MS datasets available as Frictionless Tabular Data Package^18^.

### The future is FAIR data at the source

FAIRifying data retrospectively nevertheless remains limited and challenging. Data readiness needs to begin at the source. Our exemplar approach to make these rose metabolomics datasets FAIRer is very generic, applicable to most ‘omics’ datasets and should encourage experimentalists and data scientists to capture the intent of the experimental design prospectively, by regularizing and annotating the resulting matrices in a formal way. To enable data FAIRness, we will continue contributing to a number of international efforts that work to develop guidelines, tools and services (e.g. FAIREvaluator^23^) for researchers and data stewards in the life sciences (such as the European FAIRplus, https://fairplus-project.eu, the USA National Institute of Health, https://commonfund.nih.gov/dataecosystem), and across-disciplines (including GO-FAIR, https://www.go-fair.org, and the Research Data Alliance, https://www.rd-alliance.org). We need new technological and social infrastructure, to transform the concept of data readiness into a powerful toolkit at the researchers’ fingertips, to realize FAIR data.
